# Deconvolution of RNA-Seq Analysis of Hyperbaric Oxygen-Treated Mice Lungs Reveals Mesenchymal Cell Subtype Changes

**DOI:** 10.3390/ijms21041371

**Published:** 2020-02-18

**Authors:** Yuan Yuan, Yilu Zhou, Yali Li, Charlotte Hill, Rob M. Ewing, Mark G. Jones, Donna E. Davies, Zhenglin Jiang, Yihua Wang

**Affiliations:** 1 Department of Neurophysiology and Neuropharmacology, Institute of Special Environmental Medicine and Co-innovation Center of Neuroregeneration, Nantong University, Nantong 226019, Jiangsu, China; 2 Biological Sciences, Faculty of Environmental and Life Sciences, University of Southampton, Southampton, SO17 1BJ, UK; 3 Institute for Life Sciences, University of Southampton, Southampton, SO17 1BJ, UK; 4 Clinical and Experimental Sciences, Faculty of Medicine, University of Southampton, Southampton, SO16 6YD, UK; 5 NIHR Southampton Biomedical Research Centre, University Hospital Southampton, Southampton, SO16 6YD, UK

**Keywords:** Hyperbaric oxygen, lung, RNA-seq, CIBERSORTx

## Abstract

Hyperbaric oxygen (HBO) is widely applied to treat several hypoxia-related diseases. Previous studies have focused on the immediate effect of HBO-exposure induced oxidative stress on the lungs, but knowledge regarding the chronic effects from repetitive HBO exposure is limited, especially at the gene expression level. We found that repetitive HBO exposure did not alter the morphology of murine lungs. However, by deconvolution of RNA-seq from those mice lungs using CIBERSORTx and the expression profile matrices of 8 mesenchymal cell subtypes obtained from bleomycin-treated mouse lungs, we identify several mesenchymal cell subtype changes. These include increases in *Col13a1* matrix fibroblasts, mesenchymal progenitors and mesothelial cell populations and decreases in lipofibroblasts, endothelial and *Pdgfrb* high cell populations. Our data suggest that repetitive HBO exposure may affect biological processes in the lungs such as response to wounding, extracellular matrix, vasculature development and immune response.

## 1 Introduction

Hyperbaric oxygen (HBO) refers to the inhalation of pure oxygen under greater than one atmosphere pressure, and is now widely used as a treatment for hypoxia-related diseases and some ischemic injuries [1]. Despite HBO being frequently administered in various clinical situations, it has several side effects, the main one being oxygen toxicity, with the risk of developing toxicity increasing with pressure and duration of treatment [1]. The lungs are the primary sites of oxygen toxicity, as a result of their direct exposure to the high partial pressure of oxygen during HBO treatment. Several conditions in pre-term and neonatal infants have been reported which are linked to hyperoxia, including hyperoxia-induced impairment of alveolarization, pulmonary inflammation, bronchopulmonary dysplasia complicated with pulmonary hypertension suggesting the developing lung is more sensitive to hyperoxia [2–6]. Conversely, the impact of hyperoxia on the adult lung appears to be relatively mild with current HBO therapies being reported to be clinically safe [7]. Previous studies suggest that HBO exposure induces oxidative stress, which activates antioxidant systems [8–11]. In addition, several conditions may benefit from the potentially protective effects of HBO exposure, including toxic-induced lung injury [12–15], smoke-induced pulmonary edema [16], hyperoxic acute lung injury [17], and acute pancreatitis-induced lung injury [18].

An extensive and unbiased study of the influence of HBO exposure on lung tissue is required, not only to validate the safety of this therapy, but also to potentially expand diseases which this treatment can be used for, such as those where hypoxia drives the pathogenesis of the disease [19–23]. Using CIBERSORTx deconvolution of RNA-seq in mice lungs exposed to HBO, we identify changes in several mesenchymal cell subtypes. These include increases in *Col13a1* matrix fibroblast, mesenchymal progenitors and mesothelial populations and decreases in lipofibroblast, endothelial and *Pdgfrb* high cell populations. This analysis is based on the profile matrix of 8 mesenchymal cell subtypes obtained from bleomycin-treated mouse lungs. Through the Gene Ontology (GO) enrichment analysis of the differentially expressed genes (DEGs) of the eight mesenchymal cell subtypes, their functions were inferred. Combining the CIBERSORTx analysis with the enrichment analysis, our data suggest that repetitive HBO exposure may affect biological processes in the lungs such as response to wounding, extracellular matrix, vasculature development and immune response.

## 2 Results

### 2.1 Repetitive HBO Exposure Has Little Impact on Mice Lung Morphology and Body Weight

**Figure 1:**
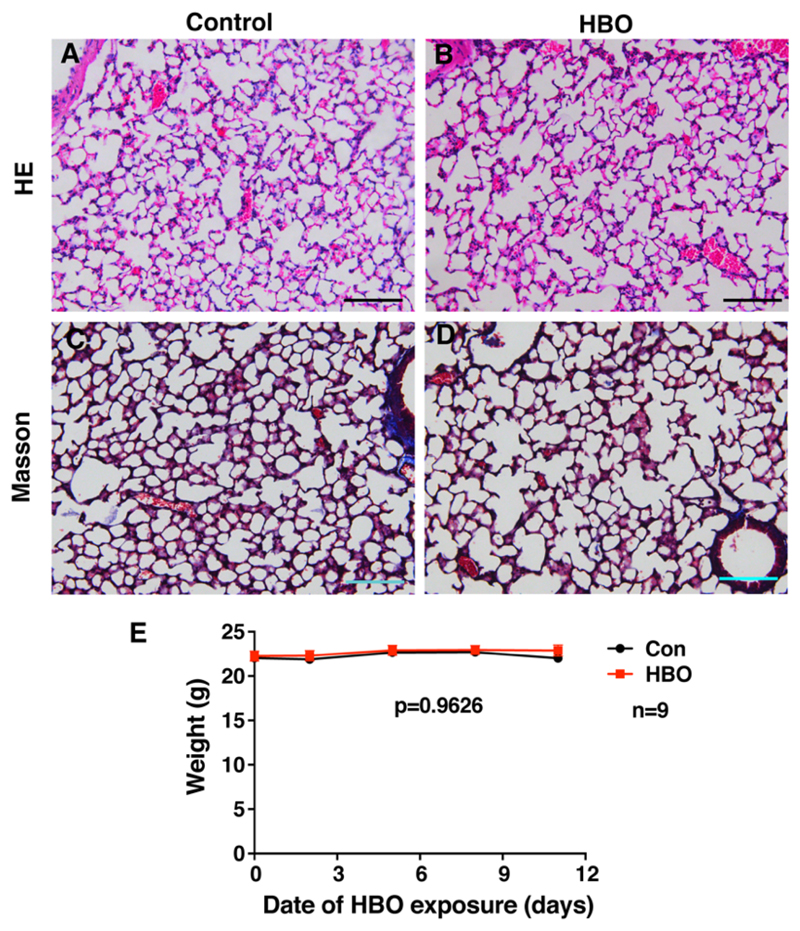
Repetitive HBO exposure had little effect on lung tissue structure and weight. (**A**) and (**B**) (top panel) show H/E staining results, (**C**) and (**D**) (bottom panel) show Masson’s trichrome staining (collagen shown in blue). (**A**) and (**C**) (left panel) show control group, while (**B**) and (**D**) (right panel) show HBO group. Scale bar represents 100 μm, *n* = 4 in control group and *n* = 5 in HBO group. (**E**) shows control (blue) and HBO-treated (red) mice weight against time, *p*-value was calculated by two-way ANOVA and the error bars represent standard deviation.

To assess the effect of repetitive HBO exposure on mice lungs, mice were exposed to 2.5 ATA (Atmosphere Absolute) HBO for 90 min, once a day, for 11 consecutive days. This protocol is consistent to those used in clinic, with the difference being a 2-day rest following 5-day exposure, due to weekends in clinical application. Mouse lungs were collected on day 12. Hematoxylin-eosin (H/E) staining of mice lungs showed little difference between the control group and HBO-treated mice, HBO-treated mice displayed well organized alveolar structure similar to control group mice (Figure 1A,B). Lung injuries can cause fibrosis, and this is characterized by the accumulation of collagen in the lungs, Masson’s trichrome staining was performed to visualize the collagen distribution. No obvious accumulation of collagens was observed in HBO-treated mice (Figure 1C,D). Mice were weighed every three days, as an indicator of the general health of the mouse, and no significant difference was observed between HBO and control group (Figure 1E).

### 2.2 Repetitive HBO Exposure Shows Mild Effects on Gene Expression in Mice Lungs

Although we did not observe changes in morphology, we postulated that the gene expression profiles of HBO treated lungs may be altered before changes in morphology and function are observed. Therefore, RNA-sequencing (RNA-seq)-based analysis was conducted to detect the effect of repetitive HBO exposure on gene expressions of lung tissues in an unbiased manner. The samples were collected at the same time point as the morphological observation to rule out the immediate effect of HBO exposure. Four samples of each group were sequenced. A total of 179,623,190 reads of the control group (44,905,797 reads per sample on average, ranging from 38,614,624 to 53,412,322 reads) and 174,456,292 reads of the HBO group (43,614,073 reads per sample on average, range from 39,350,919 to 47,038,994 reads) passed the quality control. 93.66% of overall reads were mapped to the GRCm38 reference. Using the criteria described in the methods, 16,692 genes with more than 10 counts across the eight samples were identified for further analysis.

**Figure 2:**
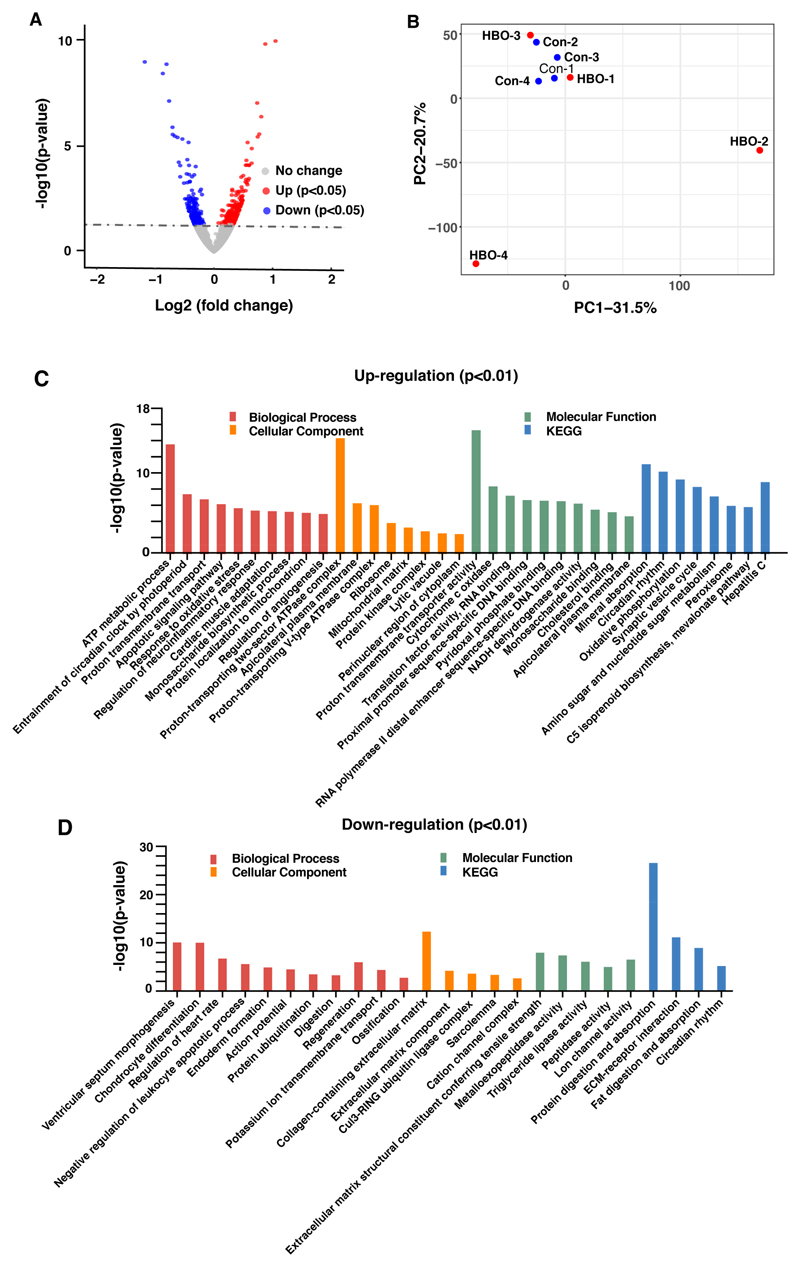
Repetitive HBO exposure had a small influence on the gene expression of lung tissues. (**A**) Volcano plot of genes using DEseq2, showing the differences in expression values between the control and HBO group. Grey dotted line indicates the threshold for *p*-value = 0.05. Blue and red points represented down-regulated and up-regulated differentially expressed genes respectively. (**B**) Principal components analysis (PCA) of transcriptomic data from each sample. The first two principal components were shown in the plot and represented 31.5% of the variation (PC1) and 20.7% of the variation (PC2). (**C**) and (**D**) GO and KEGG enrichment analysis of up-regulated (**C**) and down-regulated differentially expressed genes (*p* < 0.01). Biological processes are indicated in red, orange indicates cellular component, green shows molecular function and blue bar shows KEGG pathway.

DEGs were analyzed using DEseq2 [24]. When screened with a commonly used rigid threshold *p*adj < 0.05 (adjusted *p*-value), only 33 genes were differentially expressed, of which 15 genes were up-regulated and 18 genes were down-regulated (Table S1). Seven out of 15 genes were up-regulated more than 2-fold, including *Spink5*, *Nr1d2*, *Dbp*, *Nmrk2*, *Ddit4*, *Nr1d1*, *mt-Atp6*. 14 out of 18 genes were down-regulated more than 0.5-fold: *Ctrb1*, *Spon2*, *Pnlip*, *Cela2a*, *Cpa1*, *Igkv6-14*, *Prss2*, *Rag1*, *Try4*, *Try5*, *Ighv5-9*, *Cel*, *Ighv3-8*, *Amy2b*. Some of the abundantly expressed genes were validated through real time qPCR, and the expression change of *Ddit4*, *Spink5*, *mt-ATP6*, *mt-CO3*, *Spon2* and *Prss2* was consistent with RNA-seq results (Figure S1). When the threshold was adjusted to *p* < 0.05 (*p*-value), 636 differentially expressed genes were selected, of these 364 were up-regulated while 272 were down-regulated (Figure 2A), the differentially expressed genes are listed in Table S2.

To compare the differences between control and HBO groups, we calculated the covariance of all samples and built a matrix that represents the overall features of the data for principal components analysis (PCA). The first and second component represented 31.5% and 20.7% of the variance within the transcriptomic data respectively. In the PCA plot, HBO samples were not all well separated from control samples, indicating the difference between groups is less significant than that within groups, the influence of HBO exposure is minimal (Figure 2B).

To examine processes and pathways which may be altered upon HBO-exposure, GO and Kyoto Encyclopedia of Genes and Genomes (KEGG) enrichment analysis based on the *p* < 0.05 DEGs was conducted. Several biological processes were identified, including ATP metabolism, proton transport and response to oxidative stress. Further signaling pathways related to these processes were shown to be enriched in the up-regulated genes (Figure 2C,D), which was consistent with previous studies [9,11,25]. No other processes identified appeared to be directly related to the influence of extra oxygen and oxidative stress. In the down-regulated genes, enriched terms for cellular component classification included “collagen containing extracellular matrix” and “extracellular matrix component”, suggesting that the extracellular matrix may be affected.

Since the gene expression changes were minimal when compared the HBO exposure group and control group, gene set enrichment analysis (GSEA) [26] may be a more appropriate method to extract biological insight from the RNA-seq data. Through this analysis, biological processes like protein translation, mitochondrial respiratory chain, redox homeostasis, detoxification and immune related processes were up-regulated, while processes related to extracellular matrix were down-regulated (Table 1 and Table S3), which was consistent with GO enrichment analysis.

### 2.3 Repetitive HBO Exposure Has Little Effect on the Relative Composition of Different Cell Types of Mice Lungs

**Figure 3:**
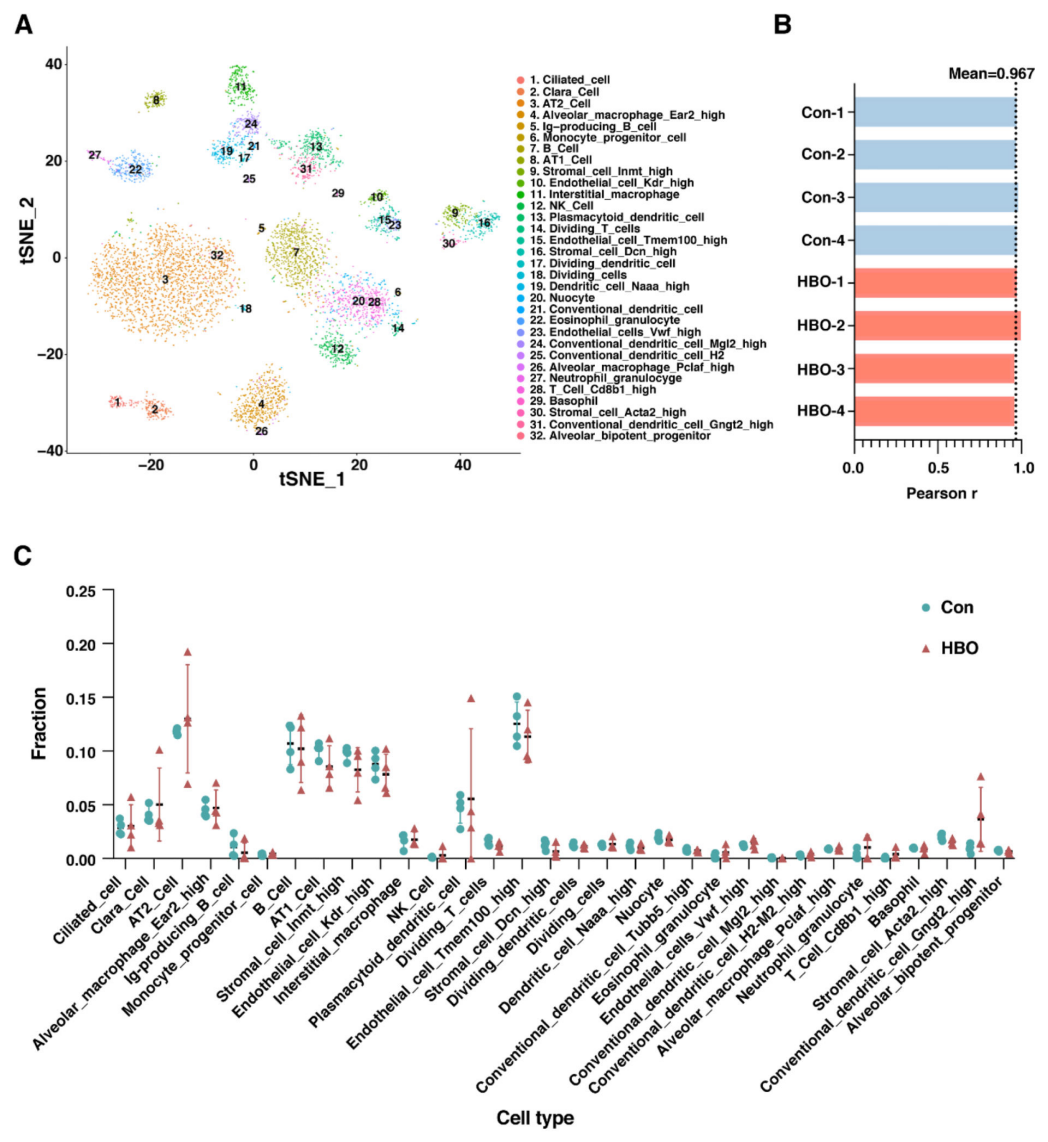
Repetitive HBO exposure had little effect on the composition of the 32 cell subtypes of lung tissue through CIBERSORTx analysis. (**A**) t-distributed stochastic neighborhood embedding (t-SNE) projection plot of the adult mouse lung scRNA-seq data from the MCA project [29]. Different colors indicate different clusters, key in figure indicates which colour corresponds to which cell type. 32 clusters identified. (**B**) Bar plot shows Pearson correlation coefficients, for control (blue) and HBO-treated (red) mice, between our transcriptome data and the scRNA-seq data. Black dotted line indicated mean Pearson correlation coefficients across all samples. (**C**) Comparisons of the ratios of the 32 cell types in control (blue) and HBO-treated (red) groups. *Y*-axis indicates the fraction of each cell type, *X*-axis are the 32 cell types. Error bars represent standard deviation.

The cellular composition of the lung tissue varies according to developmental stages, and between normal and pathological status [27]. To further analyze the influence of repetitive HBO exposure on the cell composition of lung tissue, we utilized a recently developed machine learning method called CIBERSORTx analysis [28]. The single-cell RNA sequencing (scRNA-seq) data of adult mouse lung from the Mouse Cell Atlas (MCA) project [29] were used to determine the gene expression signature of different cell types, in which 32 cell types were classified. We successfully reconstructed the cell clusters to be consistent with those reported [29], which was displayed by cell distributions according to their expression profile in two-dimensions by t-distributed stochastic neighborhood embedding (t-SNE) projections (Figure 3A). We demonstrated that the parameters we used were consistent with the original findings. Furthermore, the mean Pearson *r* value was 0.967 when comparing our transcriptome data with the scRNA-seq data (Figure 3B), demonstrating a good correlation and confidence of the analysis. Transcriptome data were then dissected by CIBERSORTx and we found there was no statistically significant difference in the ratio of any type of the cells (Figure 3C).

### 2.4 Deconvolution Based Upon Fibrotic Lung Cell Profiles Reveals Mesenchymal Cell Subtype Changes

**Figure 4:**
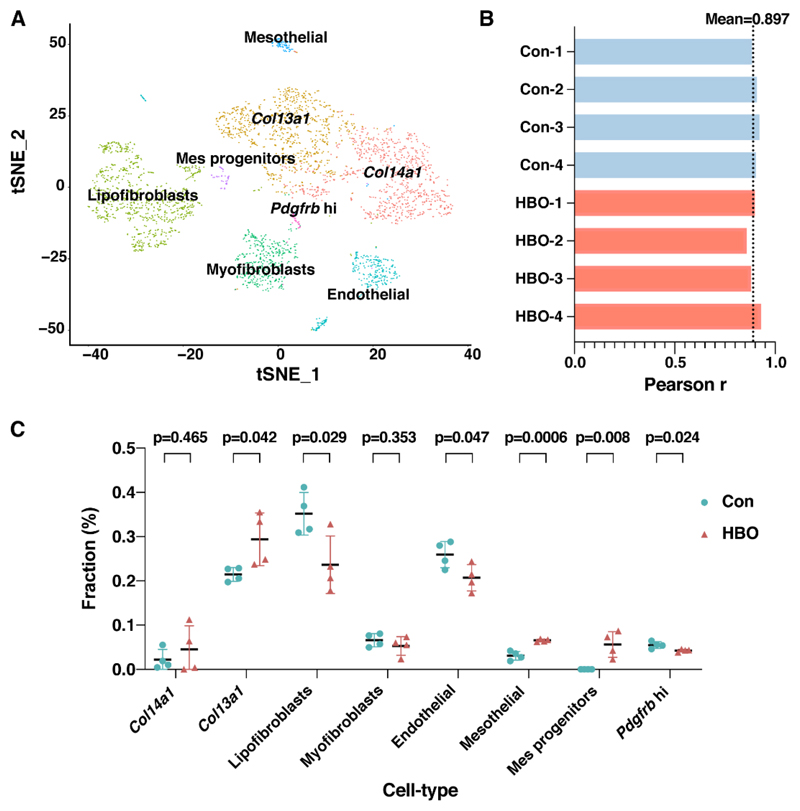
Repetitive HBO exposure affected the composition of the 8 mesenchymal cell subtypes. (**A**) t-distributed stochastic neighborhood embedding (t-SNE) projection plot of the fibrotic mouse lung scRNA-seq data [32]. Different colors indicate different clusters, as indicated by text over each cluster. Eight clusters were identified. (**B**) Bar plot represented Pearson correlation coefficients, for control (blue) and HBO-treated (red) mice, between our RNA-seq data and the scRNA-seq data. Black dotted line indicated the mean Pearson correlation coefficients across all samples. (**C**) Comparisons of the ratios of the 8 mesenchymal cell types in control (blue) and HBO-treated (red) groups. *Y*-axis indicates the fraction of each cell type, *X*-axis are the eight cell types. Error bars represent standard deviation.

Repetitive micro-injuries to lung as well as abnormal repair may result in extracellular matrix deposition in damaged lung foci and this can result in the development of pulmonary fibrosis [30,31]. Mesenchymal cells are understood to play a major role, as the origins of secretary collagen and thus be key in disease pathogenesis [30,31]. Given the fact that HBO-induced oxidative stress may cause minor injury to the lungs, we utilized another scRNA-seq dataset which focused on mouse lung mesenchymal cells, from uninjured and bleomycin-treated mouse lungs, classified and defined six mesenchymal cell types in the normal lung and seven in the fibrotic lung [32]. To determine the composition of the mesenchymal cells in the transcriptome data of lung tissues in HBO exposure and control groups, data were deconvoluted using CIBERSORTx analysis. We first reconstructed the eight cell clusters identified in the study [32] (Figure 4A), based on the scRNA-seq data of the seven subtypes of fibroblast as well as endothelial cells from fibrotic lung. Then by CIBERSORTx analysis, the transcriptome data of lung tissues in HBO exposure and control groups were dissected to determine the composition of the mesenchymal cells. Mean Pearson *r* value of our dataset and the scRNA-seq data was 0.897, suggesting a high confidence level of this analysis (Figure 4B). The inferred ratios of 6 out of 8 the cell types were found to be statistically different (*p* < 0.05) in the two groups. Among these, the ratios of *Col13a1* matrix fibroblast, mesenchymal progenitor and mesothelial were up-regulated, while those of lipofibroblast, mesothelial, and *Pdgfrb* high cells were down-regulated when comparing HBO to the control group (Figure 4C).

### 2.5 GO Enrichment Analysis Identifies Different Roles of the Eight Mesenchymal Cell Types

To identify functional consequence of the changes in the mesenchymal cell populations following repetitive HBO exposure, GO enrichment analysis of biological process using the DEGs in each of the mesenchymal cell types was performed.

**Figure 5:**
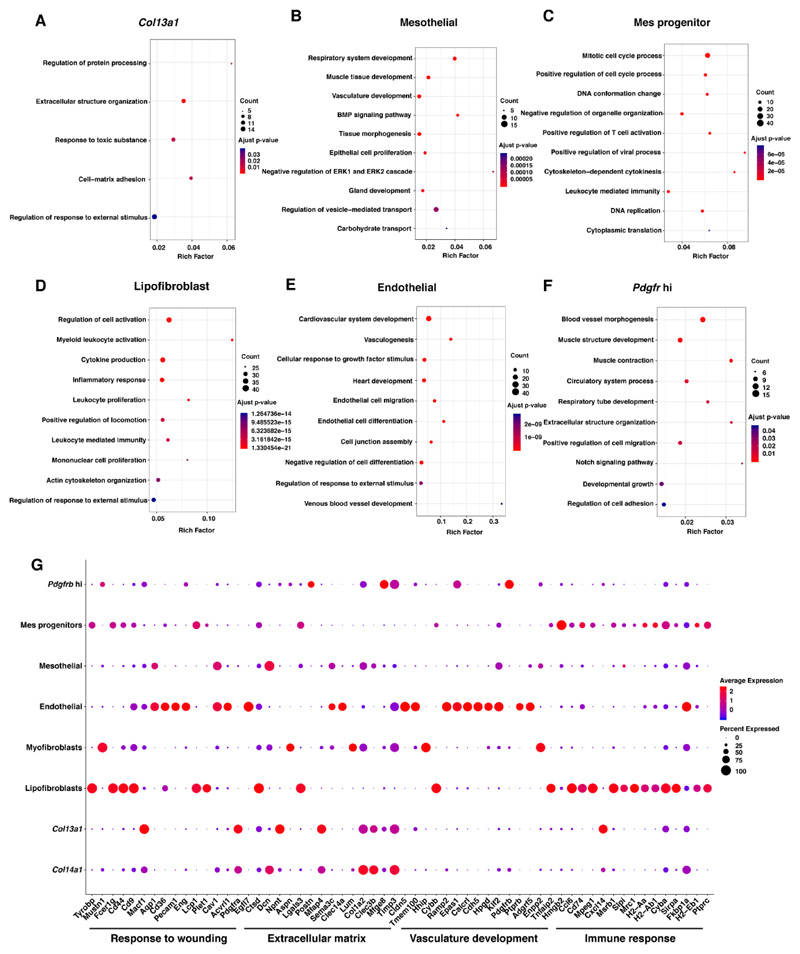
GO enrichment analysis of the differentially expressed genes in each cell type to predict the role of the different mesenchymal cell types. (**A**–**F**) Functional enrichment analysis with GO Biological Processes was performed using Metascape with all differentially expressed biomarkers in each cell population. Dot size represents the number of genes involved in the process, and dot color represents the adjusted *p*-value of the process, as described in the key. (**G**) Expression of selected genes involved in response to wounding, extracellular matrix, vasculature development and immune response in *Pdgfrb* hi, mes progenitors, mesothelial, endothelial, myofibroblasts, lipofibroblasts, *Col13a1* and *Col14a1* matrix cells. Dot size represented the percentage of cells in the cell population expressing a gene, and dot color represents the average expression level for the gene in the cell population as described by the key.

Several biological processes were identified; we focused on the non-redundant top 10 GO terms enriched (*q* < 0.01) in the biological process (all the enriched terms were shown if the number of enriched terms was less than 10) (Figure 5A–F and Figure S2). The full enrichment results are shown in Table S4–S11. Among the up-regulated cell types, the distinct expression genes in *Col13a1* matrix fibroblasts were enriched in the terms “response to toxic substance”, “extracellular structure organization” and “cell matrix adhesion” (Figure 5A). However, the term “response to toxic substance” was not enriched in *Col14a1* matrix fibroblast (Figure S2A), which is another type of *Col1a1* high expressing matrix fibroblast, and its abundance was not changed after repetitive HBO exposure. GO terms enriched in mesothelial were mainly related to development (Figure 5B), and those in mes progenitor, the potential mesenchymal progenitors, were related to cell proliferation (Figure 5C).

On the other hand, among the down-regulated cell types, the enriched GO terms in the lipofibroblasts were focused on the immune response (Figure 5D). Previously, lipofibroblasts have been reported to be involved in alveolar development, regeneration, surfactant synthesis and vitamin A (retinoic acid) storage [33], and contribute to the alveolar epithelial cell II (AECII) stem cell niche in the adult mouse lung [34]. The enrichment result in endothelial cells was mainly related to vasculature development (Figure 5E), which is consistent with the knowledge of endothelial cells as vasculature forming cells [35]. Several varied terms were identified among the enriched biological processes of *Pdgfrb* high (*Pdgfrb* hi) cells, suggesting that this cell type is a transitional cell type. *Pdgfrb* hi cells are reported to only be present in the fibrotic lung [32], suggesting it plays an important role during fibrotic pathogenesis.

Finally, the GO enrichment analysis showed that *Col14a1* matrix fibroblast might contribute to extracellular matrix organization and response to growth factor, while myofibroblast showed characteristics of response to fibroblast growth factor, and muscle tissue development which is consistent with previous reports [36]. Neither of the two cell types were altered after repetitive HBO exposure.

To compare the potential contribution of different cell types during fibrotic pathogenesis and response to oxygen, we selected four related biological processes and visualized the expression level of the top 15 significant DEGs of the 8 cell types annotated in these four processes (Figure 5G). We demonstrate that four cell types underlie these biological processes: lipofibroblasts, endothelial cells, and *Col13a1* and *Col14a1* matrix fibroblasts. Lipofibroblasts captured a signature related to immune response and response to wounding; endothelial cells showed high expression of genes related to vasculature development and response to wounding. Meanwhile, *Col13a1* and *Col14a1* matrix fibroblasts expressed high level of genes related to extracellular matrix and response to wounding, suggesting their correlation to these biological processes. Among them, lipofibroblast and endothelial cells were down-regulated, and *Col13a1* matrix fibroblasts were up-regulated. Together, these results suggested that repeated HBO exposure may have an influence on these four biological processes.

## 3 Discussion

According to the central dogma, changes in the gene expression profile occur earlier than those in morphology and functions [37]. Therefore, the present study was carried out to assess the non-instant effects of repetitive HBO-exposure on the gene expression profile of lung tissues through RNA-seq. Although morphologically normal, small changes in lung tissue gene expression were detected after repeated HBO exposure by RNA-seq followed by qPCR validations. Processes such as protein translation, mitochondrial respiratory chain, redox homeostasis, detoxification and immune related processes were up-regulated while that of extracellular matrix were down-regulated through GO enrichment and GSEA. It is critical to understand which cell type(s) is (are) important to mediate these molecular changes. The recently developed scRNA-seq technique can be used to delineate the contributions of different cell subtypes. Several studies based on the scRNA-seq of mouse lung tissue have already reported on the complexity of lung composition [29,32,38–40]. Alternatively, some bioinformatic methods, such as CIBERSORT, can be used to characterize cell composition of complex tissues from their bulk transcriptome data [41] and have previously been successfully used to enumerate immune subsets in the tumor microenvironment [42–47]. CIBERSORTx, an updated version of CIBERSORT, is a machine learning method that can infer cell-type-specific gene expression profiles without physical cell isolation, as well as dissect large-scale tissue data using scRNA-seq data [28]. In the present study, by deconvolution of RNA-seq from HBO-exposed mice lungs using CIBERSORTx and the expression profile matrices of eight mesenchymal cell subtypes obtained from bleomycin-treated mouse lungs [32], we identify several mesenchymal cell subtype changes. These include increases in *Col13a1* matrix fibroblasts, mesenchymal progenitors and mesothelial cell populations and decreases in lipofibroblasts, endothelial and *Pdgfrb* high cell populations.

Lung fibroblasts are a heterogeneous group of cells, producing extracellular matrices to build the lung structural framework, while also directing cell growth by cell–cell and cell–matrix contact and/or paracrine mechanisms [30,31,48]. Based on different gene expression profiles and expected functions, subsets of fibroblasts, including myofibroblasts, *Col13a1* matrix fibroblasts, *Col14a1* matrix fibroblasts, lipofibroblasts, mesenchymal progenitors, and mesothelial cells, and *Pdgfrb* hi fibroblast subpopulation, have been delineated [32].

Based on our analysis, the distinct expression genes in *Col13a1* matrix fibroblast were enriched in response to toxic substances, and this cell type expanded in fibrotic lung [32], suggesting that it was an important contributor to fibrotic pathogenesis. Its ratio increased in the HBO group suggesting that repetitive HBO exposure might induce some toxic stress. Lipofibroblasts have been reported to contribute to AECII stem cell niche [34] and we showed HBO exposure resulted in reduction of lipofibroblasts, which could be detrimental for AECII stemness maintenance. HBO treatment has been shown to alter inflammation [1]; consistent with that, we found that lipofibroblasts acquire a gene signature involved in immune response and the reduction of this cell type might cause inhibition of inflammation. Chronic wounds exist in a perpetually inflammatory state [1], as such, the inhibition of inflammation might be beneficial. A recent study showed *Pdgfrb* high cells to be responsive to fibrotic injury [32], reduced levels of these cells following HBO exposure may be protective. A decrease in endothelial cells was observed after HBO exposure, these are a major player in vasculature development. Although HBO treatment promotes neovascularization [1], this effect seems to be tissue specific, as HBO exposure may reduce the blood vessel density in some cancer models [49,50]. Furthermore, both high and low oxygen concentrations have been reported to cause pulmonary vascular remodeling in neonatal infants [5,51]. We suggest that the reduction in the endothelial cell population might be an adaptive response to extra oxygen.

Generally, we found the results from CIBERSORTx analysis, traditional DEG analysis and GSEA to be consistent. Reports have demonstrated that HBO can induce oxidative stress [25]; we found increased expression of *Ddit4* (*REDD1*), a gene induced in response to various stresses [52], the enrichment of gene sets like cell redox hemostasis and detoxification in HBO-group through GSEA, and the increase of *Col13a1* matrix fibroblasts, which capture a gene expression profile of response to toxic substance. All of which suggested repetitive HBO exposure induced stresses to lung tissue, consistent with the previous report [25]. Another example is the influence of HBO exposure on inflammation. *Spon2* (*Mindin*), which encodes an integrin ligand and pattern recognition molecule that is critical for inflammatory cell recruitment [53,54], was down-regulated by about 50% in the HBO group. *Igkv6-14*, *Ighv5-9*, and *Ighv3-8*, which all encode an immunoglobulin component (through searching in NCBI gene database), were down-regulated significantly in HBO group. *Spink5*, which encodes the serine protease inhibitor Lympho-epithelial Kazal-type inhibitor (LEKTI) and plays an anti-inflammatory role [55], is increased in the HBO group. All of these changes in gene expression suggest that HBO exposure inhibits inflammation. The gene set of “negative regulation of immune response” was enriched in HBO group suggesting an inflammation inhibition effect too. The immune inhibition information we get from the DEG analysis and GSEA are consistent with the reduction in lipofibroblasts, which characteristically show activation of inflammation, and consistent with the reports that HBO exposure attenuate inflammation in a few of conditions [56–61].

However, there are also some inconsistencies among CIBERSORTx, DEG and GSEA analysis. For example, the GO term “extracellular matrix” was enriched in the downregulated genes through GSEA; however, *Col13a1* matrix fibroblast was upregulated in the HBO group, while *Col14a1* matrix fibroblast remains unchanged, the total ratio of *Col13a1* and *Col14a1* matrix fibroblast increased. Both of them are cell types expressing signature genes associated with extracellular matrix and cell adhesion [32], suggesting that the extracellular matrix was upregulated. Several studies have reported that HBO promotes fibroblast proliferation and/or extracellular matrix synthesis in vitro and in vivo [1,62–65]. In addition, the effect of HBO on angiogenesis has been reported during HBO treatment of chronic wounding and tumor tissue, although its effect is context-dependent [1,49,50]. This was evidenced by CIBERSORTx analysis, but not through DEG and GSEA analysis. More importantly, CIBERSORTx analysis provides information on potential effects of fibroblast differentiation. Compared to other deconvolution methods, one advantage of CIBERSORTx is that it considers and removes the excessive technical variation in signature matrices from single-cell references and bulk RNA expression [66]. Luca et al. [67] applied CIBERSORTx to impute cell type-specific gene expression profiles of cancer cells in The Cancer Genome Atlas (TCGA). They successfully identified stable transcriptional states of 12 cell types, almost all of them can be validated in published single-cell datasets, revealing a comprehensive insight of cellular states in the tumor microenvironment. Shi et al. [68] showed overall consistency between the result of CIBERSORTx and their novel probabilistic deconvolution model which used to predict the contribution of exosomal sources on the tissue microenvironment. The accuracy of deconvolution predictions was further validated at both the pre- and on- treatment time points. However, since the studies on the heterogeneity of fibroblast subtypes in mouse lung are recently emerging, our understanding of fibroblast subpopulations, of cell markers at the protein level, their localization in the lung, signaling programs, and functional significance are limited. Further identification of ‘‘pathogenic’’ fibroblast subpopulations in lung disease will enable us to understand the precise effects of HBO on lung functions.

## 4 Materials and Methods

### 4.1 Animals

Six- to eight-week-old male C57BL/6 mice (20–25 g) were provided by the Animal Center of Nantong University. Mice were raised under a 12 h light/dark cycle, and normal diet and water were provided ad libitum throughout the study. During the study, the mice were weighed every third day. All the animals were provided by the Experimental Animal Center of Nantong University (Institutional license: SYXK(SU)-2012-0030). All procedures involving animals were approved by the Institutional Animal Care and Use Committee (IACUC) of Nantong University (NTU), Nantong, Jiangsu Province, China (approval number: 20140901-001).

### 4.2 HBO Exposure

For hyperbaric oxygen exposure, a hyperbaric chamber designed for small animal research was used. The exposure regimen consisted of a 5-min pressure ramp-up to 2.5 ATA (1.5 atm) in a 100% O_2_ environment, sustained for 90 min at this pressure, and followed by a 5-min decompression phase. To maintain a high concentration of oxygen, the chamber was flushed twice for 5 min with pure oxygen before HBO exposure. The concentrations of oxygen and carbon dioxide were monitored in real time by SDA oxygen and carbon dioxide monitors (ANALOX, North Yorkshire, England), respectively. Twenty mice were randomized into control and HBO groups. The mice in the HBO group were subjected to the hyperbaric oxygen exposure once a day for 11 consecutive days, while the mice in the control group were placed in the chamber for the same duration without pure oxygen pressurization.

### 4.3 Morphological Examination

Twenty-four hours after the last HBO exposure, the mice of both groups were sacrificed under anesthesia with composited anesthetic (with 257 mM chloral hydrate, 176 mM magnesium sulfate, 36 mM pentobarbital sodium, 14.25% ethanol, and 33.8% propylene glycol), and the whole lung tissues were collected. The lung tissues were fixed in 4% paraformaldehyde and dehydrated through gradient concentrations of ethanol. The fixed lung tissues were embedded in paraffin, sequentially sectioned into 5 μm sections, and then stained with H/E solution to observe the morphology of lung tissue, and Masson’s trichrome staining to detect the presence of collagen. The selected sections were viewed and imaged using a Leica DM4000B microscope (Wetzlar, Germany).

### 4.4 RNA Isolation, Library Construction and Sequencing

RNA was isolated with Trizol (Invitrogen, Carlsbad, California, USA). A total amount of 3 μg RNA per sample was used as input material for library construction. Sequencing libraries were generated using NEBNext^®^ UltraTM RNA Library Prep Kit for Illumina^®^ (NEB, Ipswich, Massachusetts, USA) following manufacturer’s instruction. Libraries were pooled in equimolar and sequenced using the paired-end strategy (2 × 150) on the Illumina NovaSeq 6000 platform following the standard protocols. RNA-seq data have been deposited in the Gene Expression Omnibus (GEO) database (accession code GSE143348).

### 4.5 RNA-Seq Data Analysis

Quality control of RNA-seq raw paired data was performed using FastQC (http://www.bioinformatics.babraham.ac.uk/projects/fastqc) and MultiQC [69]. Trim Galore (https://github.com/FelixKrueger/TrimGalore) was used to trim adapters, reads with low quality (<30) and short length (<30 bp). RNA-seq reads were mapped to Mus *musculus* genome Ensembl GRCm38 using Hisat2 [70] (version 2.1.0) with default codes. Sam files were transformed into bam files using samtools [71] (version 1.9). The read counts of each genes were summarized using featureCounts [72] (version 1.6.5). Raw read counts were imported into R studio (version 3.6.1) and analyzed by using R package of DESeq2 [24] (version 1.26.0). Transcripts with low abundance (under 10 count across all samples) were eliminated. Genes with false discovery rate (FDR) *p*-value < 0.05 adjusted by using Benjamini–Hochberg (BH) method were considered as differentially expressed genes (DEGs).

### 4.6 Real-Time qPCR Analysis

Real-time qPCR was done to validate the RNA-seq results. Total RNA was reverse transcribed through HiScript III RT SuperMix for qPCR (+ gDNA wiper) (Vazyme, Nanjing, China), and the qPCR was done with ChamQ Universal SYBR qPCR Master Mix (Vazyme, Nanjing, China). Primers used were as follow:

Ddit4-F: CAAGGCAAGAGCTGCCATAG,

Ddit4-R: CCGGTACTTAGCGTCAGGG;

Spink5-F: ACACTGACGTGTCCCAAAGG,

Spink5-R: GGTGCTCCTTGTTCTAGTGCT;

mt-ATP6-F: TCACTTGCCCACTTCCTTCC,

mt-ATP6-R: TTAGCTGTAAGCCGGACTGC;

mt-CO3-F: ATGGCATTAGCAGTCCGGC,

mt-CO3-R: TGTAATGGTAGCTGTTGGTGGG;

Axin2-F: TCAGTAACAGCCCAAGAACCG,

Axin2-R: CCTCCTCTCTTTTACAGCAAAGC;

Spon2-F: TGCTTCCTTGTCTCAAGCCC,

Spon2-R: TCCATCACCTGGCAAAAGCG;

Prss2-F: CATGGACCCTGGACAATGACATC,

Prss2-R: GTTGTTCACACCATTGCTGAGG.

β-actin was used as the endogenous control, and its primers were:

β-actin-F: ACACCCGCCACCAGTTC,

β-actin-R: TACAGCCCGGGGAGCAT.

### 4.7 Single-Cell Data Analysis

Single-cell datasets were downloaded from GEO (Accessions GSE108097 [29] and GSE104154 [32]). We used Seurat [73] (version 3.0) to filter cells, select the number of principal components, perform t-distributed stochastic neighbor embedding (t-SNE) reduction according to the description in the original papers, ensuring the same classification of cell types. Numerous biomarkers of defining clusters were identified through FindVariableGenes function.

### 4.8 CIBERSORTx Analysis

We used CIBERSORTx [28] to estimate the percentage of various cell populations in each bulk-RNA sample. Signature matrix of each cell type was generated following the default settings. The deconvolution of bulk RNA-seq using the signature matrix with S-batch correction removed variances between different sequence platforms. The permutation value for obtaining robust statistical result was set as 1000. Two-tailed, unpaired Student’s t-test was used to compare significant differences in abundances of member cell types between two conditions. *p*-values were adjusted for multiple testing using Benjamini-Hochberg method.

### 4.9 Pathway Enrichment Analysis

Gene ontology and KEGG pathway enrichment analysis were generated through Metascape [74] website (http://metascape.org). Parameters were set as five minimum overlap genes, *p* < 0.05 and 1.5 minimum enrichment factor. GSEA (gene set enrichment analysis) [26] was performed using GSEA software (the Broad Institute platform, version 4.0.3) with the default settings and 1000 gene set permutations. It was performed on normalized counts when comparing HBO samples versus control samples. All gene sets with an FDR (false discovery rate) *q*-value less than 0.1 were considered to be significant.

### 4.10 Statistical Analysis

Each experiment was repeated at least twice. Each transcriptomic condition had four replicates. Unless otherwise noted, data are presented as mean and standard deviation. Two-tailed, unpaired Student’s t-test was used to determine statistical significance through Prism 8. *p* < 0.05 was considered as statistically significant.

## 5 Conclusions

Taken together, this study suggests a greater focus may be required on the lung status when using long-term, repetitive HBO treatment especially in relation to wounding, extracellular matrix organization, vasculature development and immune responses, processes that become aberrant in the development of chronic lung diseases. Further experiments are required to confirm its impact.

## Supplementary Material

Table S11

Figure S1 qPCR validation of differential expressed genes.

Table S9

Table S8

Table S7

Table S6

Table S10

Table S4

Table S3

Table S2

Figure S2 GO enrichment analysis of the differentially expressed genes of Col14a1 matrix cells and myofibroblasts.

Table S1

Table S5

## Figures and Tables

**Table 1:** Biological processes enriched through GSEA

GO Process	NES	FDR *q*-Value
Cytosolic ribosome	2.73	<0.0001
Establishment of protein localization to endoplasmic reticulum	2.58	<0.0001
Ribosomal subunit	2.52	<0.0001
Protein localization to endoplasmic reticulum	2.51	<0.0001
Translational initiation	2.47	<0.0001
Ribosome	2.38	<0.0001
Nuclear transcribed mRNA catabolic process nonsense mediated decay	2.36	<0.0001
Cytosolic large ribosomal subunit	2.33	<0.0001
Cytosolic small ribosomal subunit	2.29	<0.0001
Structural constituent of ribosome	2.28	<0.0001
Mitochondrial electron transport NADH to ubiquinone	2.27	<0.0001
NADH dehydrogenase activity	2.26	<0.0001
NADH Dehydrogenase complex	2.26	<0.0001
Oxidoreductase activity acting on NADPH quinone or similar compound as acceptor	2.24	1.28E-04
Cytosolic part	2.25	1.37E-04
Small ribosomal subunit	2.25	1.46E-04
Oxidative phosphorylation	2.21	2.43E-04
Respiratory chain	2.20	2.86E-04
Multi organism metabolic process	2.19	4.10E-04
Large ribosomal subunit	2.19	4.32E-04
Electron transport chain	2.13	1.32E-03
NADH dehydrogenase complex assembly	2.11	1.69E-03
Interaction with symbiont	2.12	1.77E-03
Mitochondrial respiratory chain complex I assembly	2.11	1.79E-03
Mitochondrial respiratory chain complex I biogenesis	2.10	2.05E-03
RNA catabolic process	2.09	2.64E-03
Oxidoreductase activity acting on peroxide as acceptor	2.07	3.62E-03
Mitochondrial protein complex	2.07	3.64E-03
Mitochondrial respiratory chain complex assembly	2.06	3.99E-03
Nucleoside triphosphate metabolic process	2.04	5.36E-03
Proteinaceous extracellular matrix	−2.30	6.34E-04
Calcium dependent cell adhesion via plasma membrane cell adhesion molecules	−2.45	9.50E-04
Extracellular matrix component	−2.38	9.51E-04
Delayed rectifier potassium channel activity	−2.25	1.19E-03
Homophilic cell adhesion via plasma membrane adhesion molecules	−2.13	1.12E-02
Intestinal absorption	−2.12	1.20E-02
Basement membrane	−2.14	1.25E-02
Cell cell adhesion via plasma membrane adhesion molecules	−2.04	3.45E-02
Voltage gated potassium channel activity	−2.04	3.83E-02
Extracellular matrix	−2.01	4.87E-02

Notes: GO process: the gene set name; NES: normalized enrichment score; FDR *q*-value: false discovery rate. The significant gene sets were selected by the threshold FDR *q*-value < 0.05, and only the top 30 up-regulated gene sets were shown, while all the down-regulated gene sets were listed.

## Abbreviations

HBO: Hyperbaric oxygen
DEG: Differentially expressed gene
PCA: Principal components analysis
GSEA: gene set enrichment analysis
FDR: false discovery rate
t-SNE: t-distributed stochastic neighborhood embedding
RNA-seq: RNA-sequencing
scRNA-seq: Single cell RNA-sequencing
*padj*: adjusted *p*-value
MCA: Mouse Cell Atlas
AECII: alveolar epithelial cell II
ATA: Atmosphere Absolute
atm: atmosphere
H/E: hematoxylin-eosin
GO: Gene Ontology
KEGG: Kyoto Encyclopedia of Genes and Genomes
