# Supplementary figures and images for: Deconvolution of RNA-Seq Analysis of Hyperbaric Oxygen-Treated Mice Lungs Reveals Mesenchymal Cell Subtype Changes
Source: Int J Mol Sci. 2020 Feb 18;21(4):1371. doi: 10.3390/ijms21041371 (PMC7039706; doi:10.3390/ijms21041371)

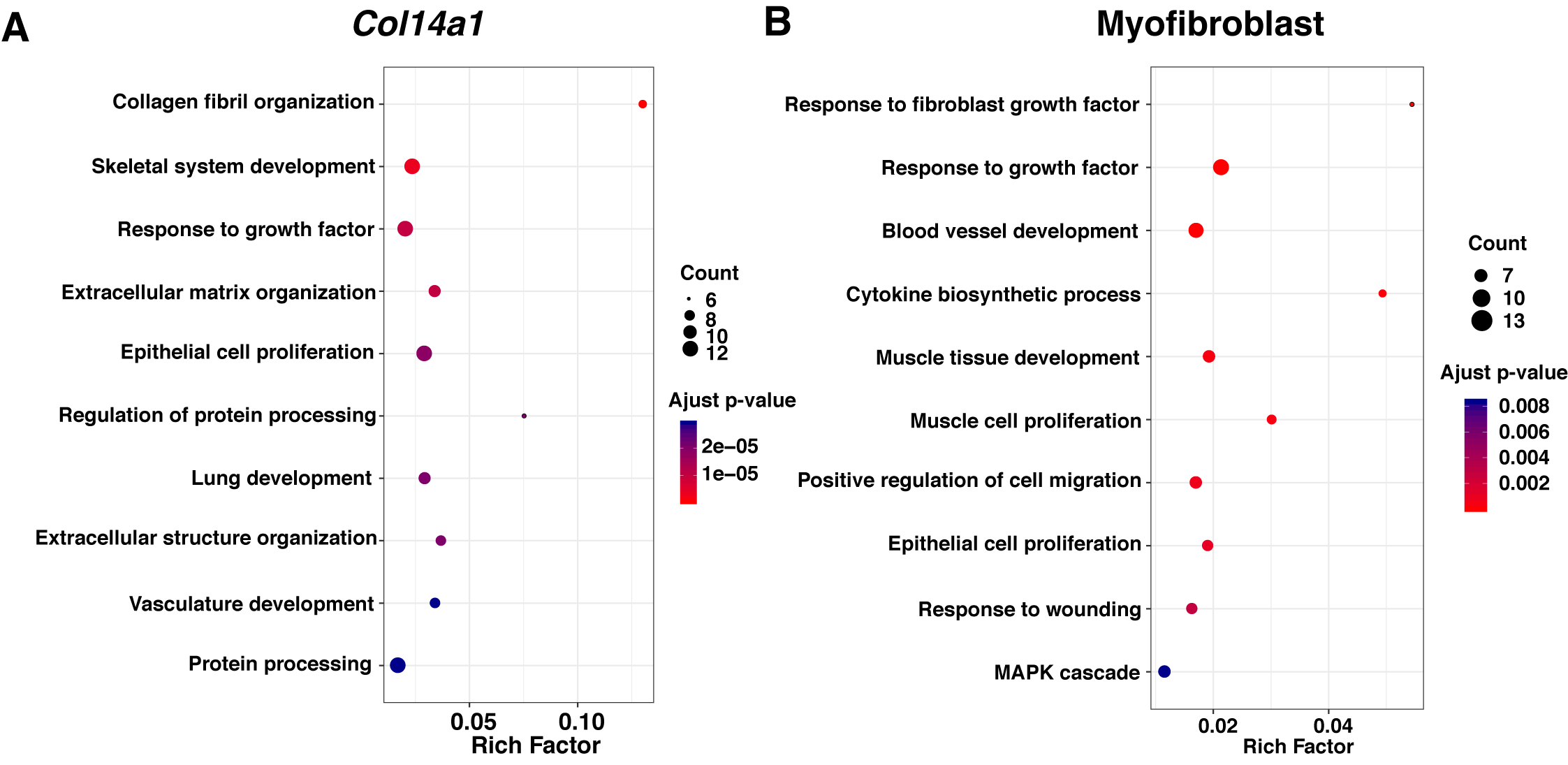

Supplement: Supplementary file 1 [file ijms-21-01371-s001.zip › Supplementary data/Figure S2. GO enrichment anlaysis.tif]

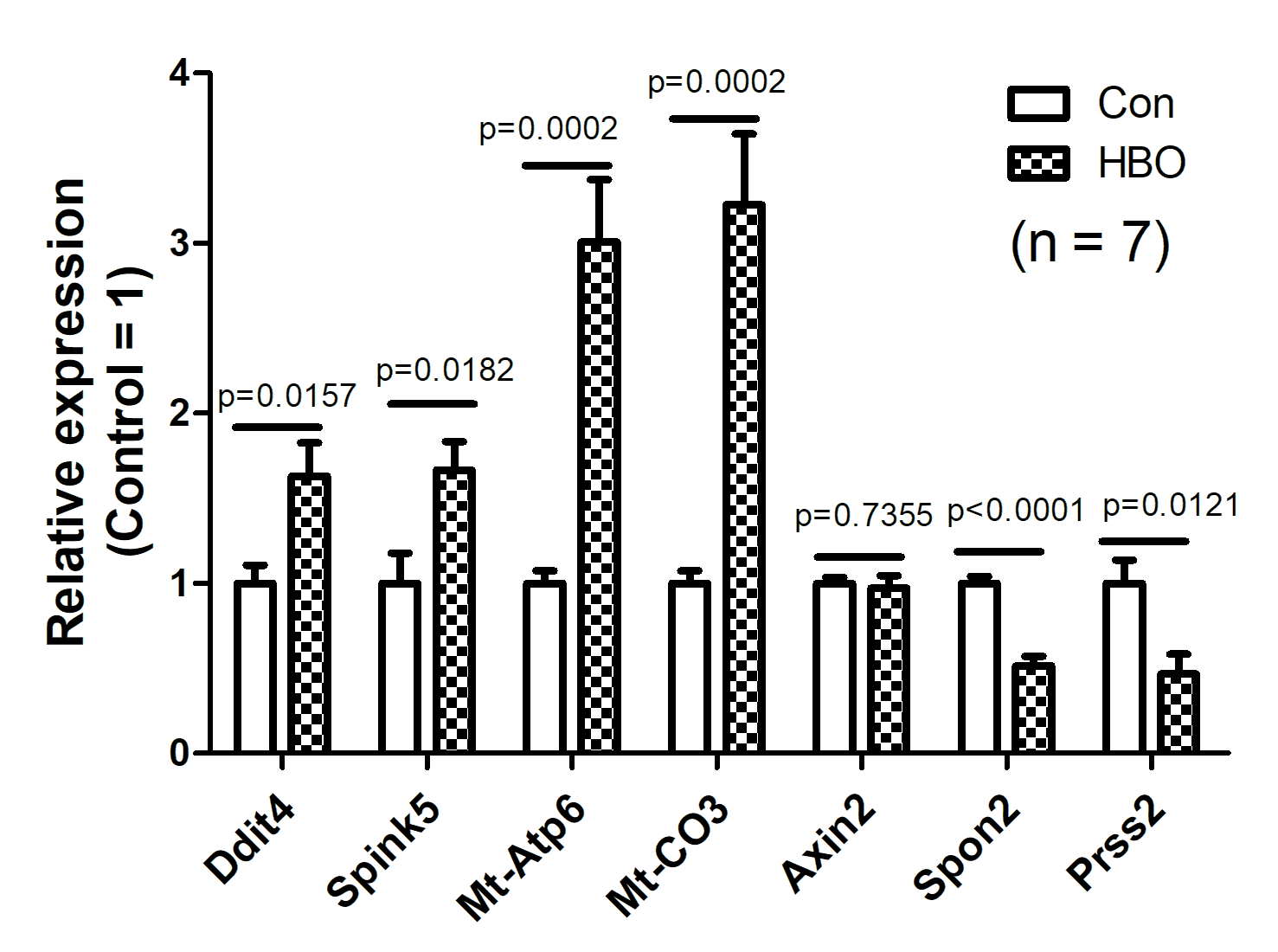

Supplement: Supplementary file 1 [file ijms-21-01371-s001.zip › Supplementary data/Figure S1. qPCR validation of differential expressed genes.tif]
